# Primary pleural synovial sarcoma with repeated resection leading to long‐term survival

**DOI:** 10.1002/rcr2.480

**Published:** 2019-08-20

**Authors:** Naoko Katsurada, Hisashi Ohnishi, Miho Ikeda, Naoe Jimbo, Yukihisa Hatakeyama, Kayoko Okamura

**Affiliations:** ^1^ Division of Respiratory Medicine, Department of Internal Medicine Kobe University Graduate School of Medicine Kobe Japan; ^2^ Department of Respiratory Medicine Akashi Medical Center Akashi Japan; ^3^ Department of Diagnostic Pathology Kobe University Graduate School of Medicine Kobe Japan

**Keywords:** Primary pleural synovial sarcoma, repeated recurrence, repeated surgery, spontaneous regression

## Abstract

Primary pleural synovial sarcoma is a malignant tumour and thought to be more aggressive than synovial sarcoma which occurs in extremities. Its treatment strategy has not been fully established because of its rarity. We report a primary pleural synovial sarcoma case which achieved a long‐term survival with repeated surgery of recurrent pleural tumour. A 39‐year‐old man presented with a gradually enlarged tumour in the left hemithorax. The tumour was resected and diagnosed as primary pleural synovial sarcoma. The tumour was slowly growing and repeatedly recurrent in the left pleura. The surgical resections for the recurrent tumours were performed 6 years and 11 years after the initial surgery. Intriguingly, recurrent tumour which developed after second surgery exhibited temporally spontaneous regression. Our patient remains alive 12 years after the initial surgery. Repeated resection of metastatic lesion can achieve long survival in primary pleural synovial sarcoma.

## Introduction

Synovial sarcoma is a soft tissue malignant tumour and the majority of that occur in extremities. Primary pleural synovial sarcoma is extremely uncommon and its therapeutic strategy has not been clearly established 1. Here, we report the case of a patient with primary pleural synovial sarcoma who had repeated surgery for ipsilateral pleural recurrent lesions and remains alive 12 years after the initial surgery.

## Case Report

A 39‐year‐old man was referred to our hospital for investigation of a mass detected by chest X‐ray. A chest computed tomography (CT) scan revealed pleura‐based opacity in the left hemithorax. The patient had no history of any serious illnesses. His laboratory findings were normal. We performed an ultrasound‐guided biopsy twice, but no definitive diagnosis was obtained. Follow‐up CT at 8 months after the second biopsy showed progressive enlargement of the lesion (Fig. 2A). We performed a third biopsy, and pathological examination revealed proliferation of spindle‐shaped malignant tumour cells. Chest and abdominal CT scans showed no evidence of distant metastases. We performed surgical resection of the tumour by wedge resection of the left upper lobe. The tumour was measuring 7.3 × 3.6 × 2.6 cm and pedunculated from the visceral pleura. Pathological evaluation showed dense proliferation of atypical spindle cells and high mitotic activity. Immunohistochemically, the tumour was positive for CD99, B‐cell lymphoma‐2 (bcl‐2), and epithelial membrane antigen. The synovial sarcoma translocation‐synovial sarcoma X chromosome breakpoint (*SYT‐SSX1*) fusion gene was detected by reverse transcriptase‐polymerase chain reaction, so a diagnosis of primary pleural synovial sarcoma was made.

**Figure 1:**
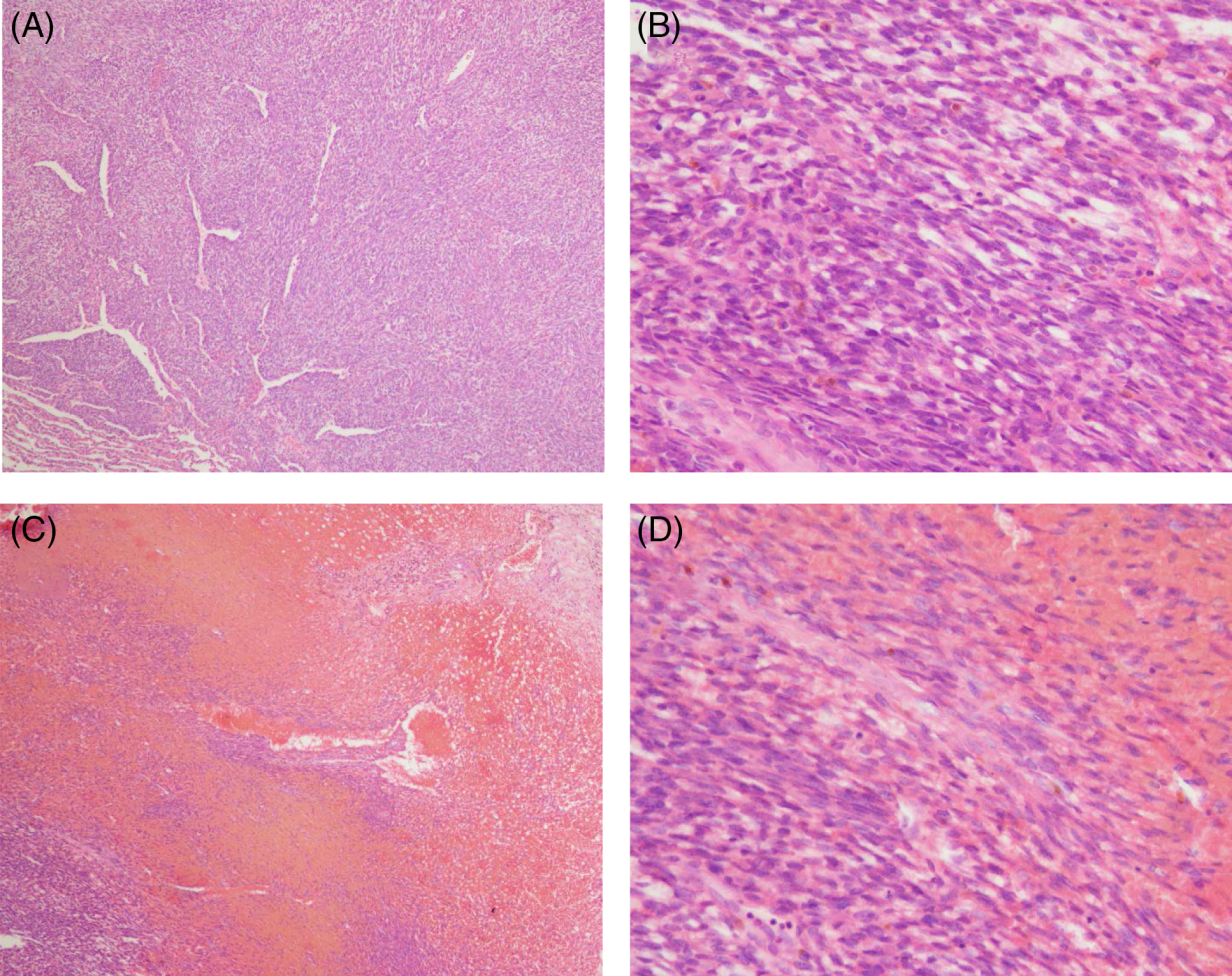
Haematoxylin and eosin staining of the recurrent tumour which resected at the second surgery showed dense proliferation of atypical spindle cells with absence of haemorrhage (A, ×4; B, ×200). Haematoxylin and eosin staining of the recurrent tumour which resected at the third surgery showed composition of small number of spindle tumour cells in massive haemorrhage with a small portion of fibrotic changes (C, ×40; D, ×200).

**Figure 2:**
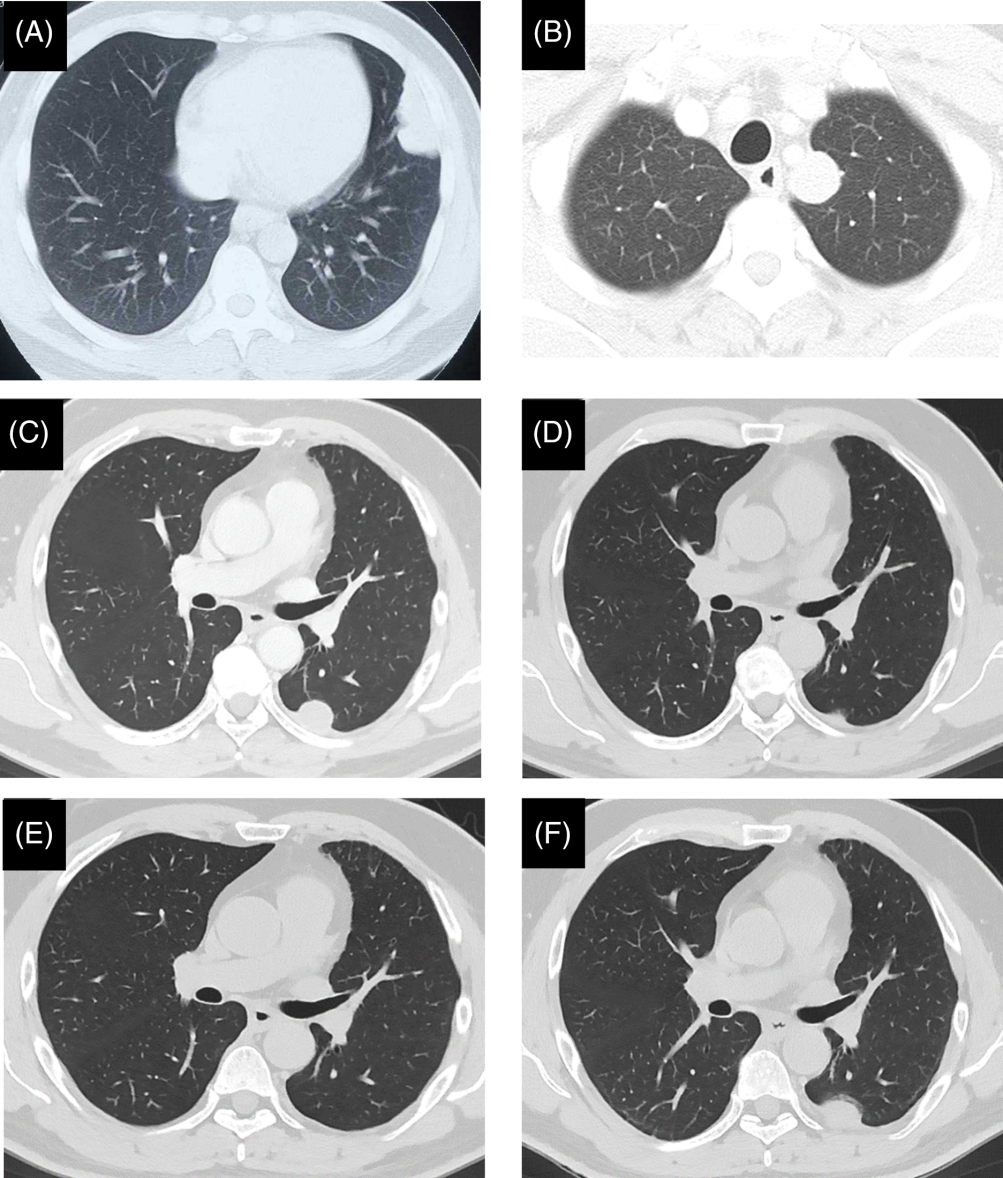
Chest computed tomography at the time of the first surgery revealed a nodule arising in the left pleura (A). Chest computed tomography at the time of the second surgery revealed a nodule adjacent to the mediastinum in the left upper hemithorax (B). Chest computed tomography revealed a nodule arising in the pleura adjacent to the left lower lobe three years after the second surgery (C). The tumour spontaneously regressed one month after C (D) and almost disappeared three months after D (E). However, the tumour increases in size again and gradually enlarged 18 months after E (F).

Two years after the initial surgery, a CT scan revealed a tiny nodule adjacent to the mediastinum in the left upper hemithorax. The tumour gradually enlarged (Fig. 2B), and 6 years after the initial surgery, wedge resection of the left upper lobe was performed again. The tumour measured 2.7 × 2.2 × 0.9 cm and arose from the visceral pleura. Pathological examination revealed spindle cell proliferation (Fig. 1A, B). The finding was compatible with recurrence of synovial sarcoma. Three years after the second surgery, the patient had left back pain and a CT scan revealed a nodule adjacent to the mediastinum in the left lower hemithorax (Fig. 2C). The tumour regressed spontaneously after 1 month (Fig. 2D) and almost disappeared after 3 months (Fig. 2E). However, the tumour showed regrowth after 6 months and gradually enlarged (Fig. 2F). Five years after the second surgery, we performed partial pleurectomy with partial resection of the involved lung and periosteum, and no tumour dissemination was observed. The tumour measured 7.3 × 5.0 × 1.0 cm and arose from the parietal pleura. Pathological evaluation showed that the tumour was composed of a small number of spindle tumour cells with massive haemorrhage and a small amount of fibrotic changes (Fig. 1C, D). The diagnosis was recurrence of synovial sarcoma. There was no sign of recurrence at the 1‐year follow‐up.

## Discussion

We herein described a case of primary pleural synovial sarcoma that grew slowly and exhibited late recurrence and temporally spontaneous regression of the recurrent tumour. Repeated surgical resection of the recurrent tumours contributed to long‐term survival.

Primary pleuropulmonary synovial sarcoma is thought to be more aggressive than synovial sarcoma arising from the extremities. According to a previous study, the median time to local recurrence or metastasis among patients with localized primary pleuropulmonary synovial sarcoma was 8.5 months 1. The median survival time was 14.5 months in patients with metastatic or localized primary pleuropulmonary synovial sarcoma 1. The present case involved relatively slow growth and late recurrence. Metastatic tumours repeatedly occurred in the ipsilateral pleura, which suggests that transcoelomic spread resulted in repeated recurrence.

The recurrent tumour that occurred after the second surgery spontaneously regressed. Spontaneous regression is a rare phenomenon that involves complete or partial disappearance of a tumour without treatment 2. The precise mechanism is unclear, but there are several hypotheses. The first hypothesis is that biopsy or surgical trauma of the primary tumour stimulates an immune response and causes regression of the remaining tumour. Another hypothesis is that spontaneous regression occurs when the blood supply is insufficient for tumour growth 2, 3. Only two previous case reports of primary pleuropulmonary synovial sarcoma involved spontaneous regression 3, 4. In both cases, the spontaneous regression occurred after biopsy. The authors mentioned that biopsy may stimulate the immune system 4. In the present case, biopsy of the recurrent tumours was not performed. Pathological examination of the tumour resected at the third surgery indicated massive haemorrhage. Additionally, the patient had chest pain when the second recurrent tumour was detected; tumour haemorrhage might have occurred, with the haematoma gradually becoming compacted as the blood was absorbed. The regression might be associated with the trauma of the second surgery.

The standard treatment of synovial sarcoma is wide resection combined with radiation, as appropriate. Complete tumour resection improves survival 1. Surgical resection of metastatic lesions is a good treatment option, although treatment for cases involving metastatic tumours has not been fully established. A previous report showed that metastasectomy led to a significant survival advantage, and it was more beneficial in patients with late recurrence 5. In the present case, tumour growth and recurrence were relatively slow and adequate repeated surgery for recurrent tumour may have contributed to longer survival. The efficacy of adjuvant chemotherapy for synovial sarcoma is controversial 1. We did not perform adjuvant chemotherapy because of its indolent behaviour. However, chemotherapy may be efficient in cases which had unresectable small lesions.

In conclusion, repeated surgical resection of metastatic lesions can lead to long‐term survival in patients with primary pleural synovial sarcoma.

### Disclosure Statement

Appropriate written informed consent was obtained for publication of this case report and accompanying images.
